# Clinical evaluation of percutaneous and intra-operative device closure of atrial septal defects under transesophageal echocardiographic guidance: one center experience and mid-term follow-up

**DOI:** 10.1186/s13019-020-1071-z

**Published:** 2020-01-14

**Authors:** Peng Zhu, Haifeng Qiang, Fei Liu, Peng Xie, Shaoyi Zheng, Yong Sun

**Affiliations:** 1 Department of Cardiovascular Surgery, NanFang hospital, Southern Medical University, GuangZhou, People’s Republic of China; 2 Department of Cardiovascular Surgery, Cardiovascular hospital, Xiamen University, Xiamen, People’s Republic of China

**Keywords:** Percutaneous, Atrial septal defect, Minimally invasive surgery, Device

## Abstract

**Objective:**

The percutaneous closure of a single secundum atrial septal defect (ASD) under transesophageal echocardiography guidance as an accepted alternative to the transcatheter closure with fluoroscopy has been proven. However, the technique has not been routinely used. This study was to present and share our experience in comparing the clinical outcomes of the percutaneous and intra-operative device closure (IODC) of atrial septal defects without fluoroscopy.

**Methods:**

From January 2013 to December 2016, 103 patients with maximum diameters of ASD of less than 30 mm were allocated to groups taking either the percutaneous closure of atrial septal defects approach (PASD group, *n* = 53) or the intra-operative device closure approach (IODC Group, *n* = 50). They were operated on using the minimally invasive Amplatzer duct occluder under the guidance of transesophageal echocardiography without cardiopulmonary bypass. Echocardiography was performed to obtain an en face view of the ASD and important surrounding structures before the operation. Patient characteristics, perioperative data, and follow-up data were retrospectively documented and analyzed.

**Results:**

Patient characteristics were comparable between the two groups. These were no differences in the maximum diameters of defects and the size of the occluders in each group (16.4 ± 5.3 mm vs16.4 ± 5.2 mm, *P* = 0.98; 22.4 ± 5.8 mm vs 21.3 ± 6.6 mm, *P* = 0.38). Intracardiac manipulation time was 20.72 ± 7.70 min in the PASD group and 6.01 ± 1.03 min in the IODC group (*P* < 0.001). The procedure time was 28.70 ± 10.41 min in the PASD group and 39.13 ± 6.03 min in the IODC group (*P* < 0.001). The successful closure defect was 100% in both groups when the maximum diameter of defect less than 25 mm. Four patients the PASD groups with maximum diameters between 25 mm and 30 mm were transferred to the IOCD group after unsuccessful device implantations. The total occlusion rate was 82% immediately after deployment, 98% at 3 months, and 100% at 6 months. No cardiac-related complications occurred during the follow-up period of between 3 to 65 months (mean 21.4 ± 9.8 months).

**Conclusions:**

Percutaneous device closures of Secundum atrial septal defects showed safety and high efficiency in patients under guidance by transesophageal echocardiography when compared with intra-operative device closures and are especially suited for women and children.

## Background

Atrial septal defect (ASD) is one of the most common congenital cardiac malformations [1]. The closure is routinely performed with the patient undergoing interventional transcatheter occlusion or conventional surgical repair with cardiopulmonary bypass (CPB). Although traditional open-heart surgery is safe with excellent outcomes for all types of ASD, CPB, and sternotomy are important factors that might lead to increased postoperative complications [2]. As an alternative to surgical closure, the percutaneous device closure of ASD has been used with increasing frequency in the past years [1, 3]. However, the percutaneous approach can be challenging because the patient has to undergo multiple exposures to medical radiation, which is associated with a spectrum of malignancy, especially in women and children [4].

In recent years, intra-operative device closure of ASD without CPB under the guidance of trans-esophagus echocardiography has been developed and applied clinically with good results [5]. However, this technique is performed through a small right anterolateral thoracotomy incision. This approach may be associated with postoperative pneumothorax, pain, bleeding, unsightly scars, and breast cosmetic defects. Usually, a right thorax drainage tube is needed during the procedure.

Recently, percutaneous closure of atrial septal defect without cardiopulmonary bypass under transesophageal echocardiography guidance has been reported and is being increasingly performed with excellent results [3, 6]. There is still little research on the effects of percutaneous and intra-operative device closure of ASD under transesophageal echocardiography. Therefore, in this study, we report our experience in using the percutaneous and intra-operative device to repair ASD.

## Materials and methods

### Patients’ clinical details

Between January 2013 and December 2016, 103 patients with ages from 2.5 to 63 years underwent percutaneous device closures of isolated secundum ASDs (diameter 4 mm to 25 mm, median 14.6 mm) under trans-esophagus echocardiography guidance at our institution. Each routine examination included a physical examination, an electrocardiogram, a chest X-ray, and at least two instances of transthoracic echocardiography. Blood tests were done to exclude contraindications to antiplatelet therapy. Among them, 19 children were under 10 years of age. Fifty-nine patients were symptomatic, which included palpitations, exercise intolerance, shortness of breath, and chest pain. Of these 59 patients, 9 patients had mild pulmonary hypertension (pulmonary artery systolic pressure 30–45 mmHg), and 8 patients had moderate pulmonary hypertension (pulmonary artery systolic pressure 45–75 mmHg) (Table 1).

**Table 1:** Preoperative data comparison between groups of patients

	Gourp A (*n* = 53)	Group B(*n* = 50)	*P*-valve
Sex(F/M)	12/41	11/39	0.12
Age(Y)			0.56
2.5–5	2	13	
5–10	6	11	
> 10	45^a^	26	
Weight	54.8 ± 20.7	50.8 ± 17.5	0.31
Pulmonary hypertension			0.69
mild	5	4	
moderate	4	4	
EF(%)	69.75 ± 7.43	68.23 ± 5.47	0.14

^a^include transfer into IODC group

The following including criteria were applied: 1) be of age greater than 2.5 years, 2) has a confirmed diagnosis of single ASD by transthoracic echocardiography, 3) ASD diameter is smaller than 5 mm and usually no larger than 25 mm, 4) pulmonary hypertension is no more than 75 mmHg (1 mmHg = 0.133Kpa), 5) vena cava maximum diameter must be more than 5 mm, and 6)must have a hemodynamically left-to-right shunt.

Exclusion criteria were: 1) an ostium primum defect or venous sinus ASD, 2) severe pulmonary hypertension that can lead to a right to left shunting, 3) a puncture site with infective or venous thrombus, and 4) coexisting cardiac anomalies that need to be corrected under open repair.

The Ethics Committee of Xiamen University Cardiovascular hospital approved this study before we selected the patients for the procedures. The informed consent about participating in the study was obtained from each patient or guardian who was fully informed of the available treatment options.

### Echocardiography

ASD was detected using pre-operation transthoracic echocardiography (TTE) (Vivid E9; GE Healthcare, Little Chalfont the United Kingdom) with Doppler interrogation and color from the parasternal, subxiphoid, and apical views. Transesophageal echocardiography (TEE) (IE33, Philips Healthcare, Best, The Netherlands) with a 2–0 to 7–0 MHz frequency conversion probe was used to accurately determine ASD size (the largest diameter), circumferential structures, morphologic features, and rims of the defect during the procedure.

After general anesthesia and a single lumen tracheal intubation, patients were placed in a supine position. TEE was done at the beginning of the procedure to identify TTE results and suitability for device closure. The maximum longitudinal and horizontal diameters of the defect were measured to determine the device size. Usually, the size of the device was selected by adding 4~6 mm to the maximum of the longitudinal diameter of the defect and adding 6 mm to the maximum defect diameter in the PASD group.

### Patient classification

Based on measurements of TTE before the procedure, the patients were divided into two groups. Those that weighted less than 12 kg were included in the IODC group and patients with the rim less than 5 mm away from the above structures (except inferior vena cava rim) were excluded from PASD and considered for IODC procedures.

### Device

The modified atrial septal occluder (ASO) is a self-centering device made from a nickel-titanium alloy (Shanghai Shape Memory Alloy Co. Ltd., Shanghai, China) which is suitable for transthoracic and transfemoral delivery systems. The transthoracic delivery sheath (IODC group) has been shortened, and the dilator is straight .

### Implantation technique

#### Intra-operative device closure of the atrial septal defect

**Fig. 1:**
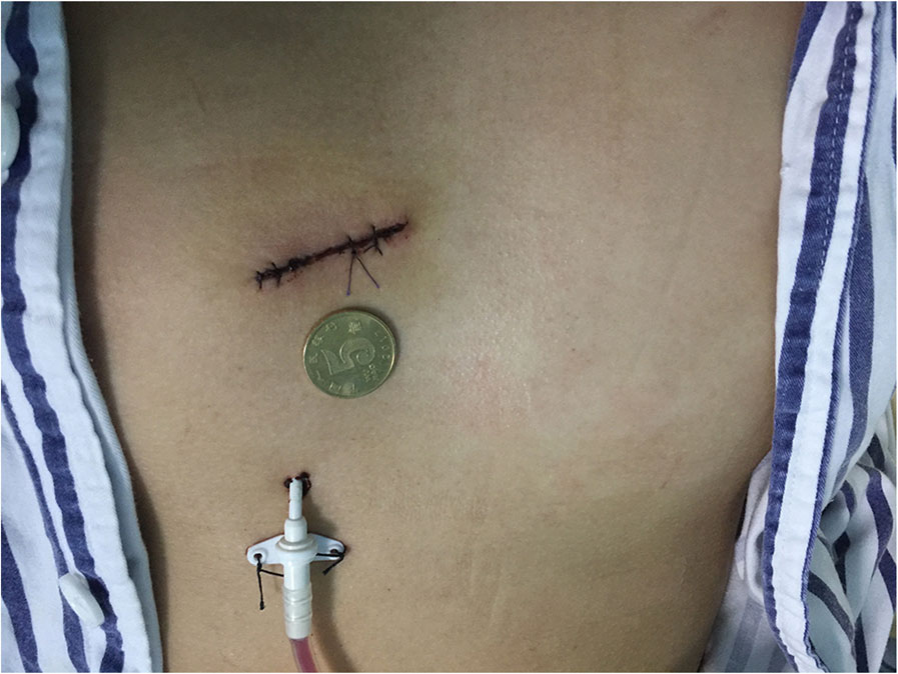
A 2.5-cm incision for IODC procedure

The minimally invasive technique of intraoperative device closure of atrial septal defect was conducted via a para-sternal approach under general anesthesia as previously described [5]. According to the preoperative X-ray, a 2.5–3 cm incision was made in the right anterior third or fourth intercostal space of the right sternal border. Under ultrasound monitoring, the best puncture point was selected at the right atrium. A dose of Heparin at 100 IU/kg and antibiotic prophylaxis was administered after incision stopped bleeding. The guide wire is sent through the defect by using ultrasound-guided modified Seldinger’s technique after a purse-string suture was placed around that point. Then the selected Amplatze septal occluder was loaded into the short delivery sheath with the tip outside. Under TEE guidance, the left side of the disc was extruded first, and slowly pulled back the delivery sheath. The left disc parallel to the atrial septum was adjusted and the right-side disc was then released. After the occluder was fully released, a pull-and-drew motion was then applied to test its stability. The occluder position and residual shunts were detected again by TEE. Finally, the mini thoracotomy was closed with a small drainage tube after making sure there is no bleeding (Fig. 1).

#### Percutaneous transvenous catheter-based device closure of the atrial septal defect

**Fig. 2:**
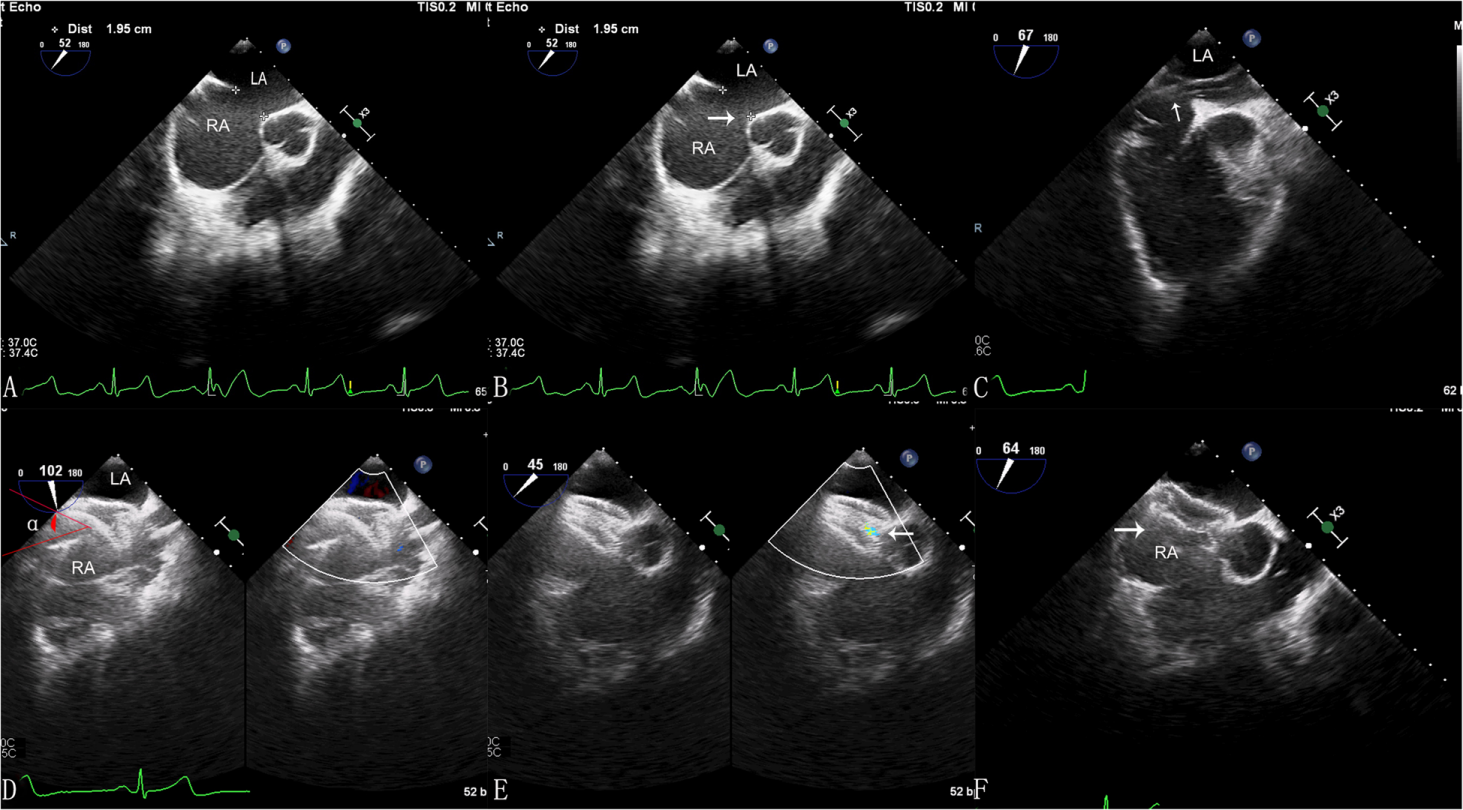
Two-dimensional transoesophageal echocardiography(TEE) imaging (**a-f**). **a** Two-dimensional image shows a larger defect. **b** There is not well rim around the defects(white arrow). **c** The delivery catheters crossing the defect. **d** The delivery catheters and atrial septal form an angle(α°). **e** A mild residual shunt(white arrow) after completely released device. **f** The occlusion is finished and the device keep perfect form(white arrow).LA:left atrium; RA:right atrium

A simplified method for atrial septal defect closure was performed through inferior the vena cava with a femoral approach [7]. The patient in supine position was placed under general anesthesia, and the femoral vein was punctured to create a device delivery track. Heparin at a dose of 100 IU/kg and the antibiotic prophylaxis was given routinely. A 180 cm 0.035-in. J-tipped super lubricated guide wire was devious to the right atrium through IVC by Seldinger’s technique. A 5 Fr MPA 2 diagnostic catheter was subsequently along the wire and keep tip against the atrial septal reach the intersection of the super vena cava and the right atrium. The guide conduit was rotated counterclockwise to move the guide wire past defect after which the catheter was extracted. This entire procedure was guided by TEE. Afterwards, the delivery sheath and the dilator were advanced along the guide wire into the left atrium through the septal defect. The Amplatzer atrial septal defect occluder is then advanced through the delivery sheath into the left atrium. Under TEE monitoring, the left disc was deployed, and the delivery sheath was pulled until the disc paralleled the atrial septal. The right disc is deployed while keeping traction on the delivery cable, and the disk was tugged to check for the positioning and hold. The occluder was released by rotating the delivery cable after no severe residual shunting was found by TTE. The sheath and the delivery cable were extracted from the right atrium, and the pressure dressing on the puncture point was made (Fig. 2).

### Patient follow-up

After the operation, heparin was given to the patients for first 24 h at 250 u/kg and aspirin at 3–4 mg/kg/day, continuing for at least 6 months. Oral antibiotics were instructed for bacterial endocarditis, to continue 48 h in PASD groups and 72 h in IODC groups.

Besides clinical examination, electrocardiography and TTE were scheduled at discharge, 3 months, 6 months, 12 months, and yearly thereafter during follow-ups. The residual shunt was defined as trivial (the jet width ≤ 1 mm), small (jet width ≤ 2 mm), moderate (jet width 2-4 mm), or large (jet width ≥ 4 mm).

### Statistical analysis

Data are expressed as mean ± SD and range. Differences between groups were compared using unpaired, 2-sided student t-tests for continuous variables. The chi-square test, or Fisher’s exact test, was used for categorical variables. A *p*-value of < 0.05 was defined as statistically significant. Data were analyzed using SPSS software (Version 22.0; SPSS Inc., Chicago, III).

## Results

### Baseline demographic and clinical characteristic

No preoperative differences were discerned between the two groups in terms of age, gender, body weight, the incidence of pulmonary arterial hypertension, diameter in defect, or heart functional status (Table 1). The percentage of patients with a history of pneumonia were normal in both groups, with no significant differences between them.

### Procedure success

As presented in Table 2, the two groups’ success showed no significant differences when ASD diameter was less than 25 mm, but the PASD group’s success decreased to 83% as the IODC groups remained at 100% success when the ASD diameter was over 25 mm. Four patients were converted to IODC after failed attempts at percutaneous transcatheter closure due to having short and soft rims of their inferior vena cava. The procedure duration was significantly shorter in the IODC group compared with the T group (*p* < 0.01), but the ICU and hospital needed much more time. Table 3 shows that the D index of the occlusion device was significantly greater in the T group than in the IODC group when the ASD diameter was smaller than 25 mm, but there were no differences between the two groups when the ASD diameter between 25 to 30 mm.

**Table 2:** Intraoperative and post-operative data comparison between groups

	Group A (*n* = 53)	Group B (*n* = 50)	*P*-value
Maximum diameter of ASD(mm)	16.4 ± 5.3	16.4 ± 5.2	0.98
Device size(mm)	22.4 ± 5.8	21.3 ± 6.6	0.38
D value	6.0 ± 1.6	4.7 ± 2.3	0.01
Rim of aortic valve (mm)	3.4 ± 1.2	2.2 ± 1.0	0.07
Table time(min)	20.72 ± 7.70	6.01 ± 1.03	0.01
Operation time(min)	28.70 ± 10.41	39.13 ± 6.03	0.01
ICU stay(hour)	4.12 ± 2.55	10.31 ± 3.64	0.01
Hospital time(d)	2.78 ± 0.9	4.7 ± 1.5	0.02
Death(%)	0	0	1
Bleeding/weigh(ml/kg)	0	12.29 ± 3.97	1
Wound infective	0	0	1
Hydrothorax	0	3	1
Residual shunt	4/50	7/50	0.52
Procedure successful	94.3(50/53)	94(47/50)	0.17
Successful 1 month(%)	98.11(52/53)	98(49/50)	0.37
Successful 3 month(%)	100	100	1

D value: difference between the device size and the maximum diameter of ASD

**Table 3:** The maximum diameter of ASD and D index

	Maximum diameter ASD < 25 mm			Maximum diameter ASD > 25 mm		
	Group A(*n* = 34)	Group B(*n* = 34)	P-index	Group A(*n* = 19)	Group B(*n* = 16)	P-index
Maximum diameter ASD(mm)	17.8 ± 3.6	18.8 ± 4.2	0.498	22.1 ± 1.8	24.0 ± 1.6	0.354
Diameter device(mm)	24.3 ± 4.6	23.1 ± 5.1	0.321	27.9 ± 1.5	28.2 ± 2.2	0.754
D index	5.8 ± 1.2	4.2 ± 2.4	0.005	5.8 ± 0.3	4.8 ± 1.6	0.639
Rim of aortic valve(mm)	3.9 ± 0.8	2.1 ± 0.4	0.26	3.2 ± 1.3	2.7 ± 1.1	0.022

*ASD* Atrial septal defect

### Complications

There were no hospital deaths. After the procedure, the trivial residual shunt was detected in 23/103 patients. Hydrothorax was noted in 4 patients by X-ray before effusions requiring drainage. Mild pericardial effusions were detected by TTE in 2/103 patients without treatment. Temporary cardiac arrhythmias were observed in 13/103 patients, but they did not require therapy except one persistent atrial fibrillation, which needed to be converted by amiodarone (Table 2). Blood transfusion was not needed in either group before discharge.

No patient had occlusion device shedding, displacement or pericardial effusion after discharge.

### Follow up

The follow-up period ranged from 3 to 65 months (mean 21.4 ± 9.8 months). Symptoms had resolved or reduced in all symptomatic patients. Pulmonary artery pressure declined or disappeared in all 16 patients who had pulmonary hypertension before the procedure. The timeline of residual shunts disappearance after the procedure is represented in Table 2. There were no residual shunts detected after 12 months follow-up. No stroke event or other major complications, including reoperations as the devices fell off, resulted in either group during the follow-up period.

## Discussion

At present, there are four standard types of surgical treatment for atrial septal defect: conventional surgery closure, thoracoscopic assistance repair, robotic assistance repair, and intra-operative device closure [8–10]. Among them, thoracoscopic assistance repair can obtain beautiful incision, but still needs extracorporeal circulation, and the possibility of blood transfusions. Compared with the thoracoscope assistance method, robot surgery has the advantages of flexibility, stability, and great mobility, but the Da Vinci system is expensive and uncommon [11].

Using the Amplazer device for ASDs has been as an accepted alternative to cardiac surgery in many centers since the first clinical care was reported in 1976 [12]. Currently, the occlusion technique is divided into three types: Classic percutaneous ASD occlusion under fluoroscopy, percutaneous procedure under ultrasound-guidance, and intra-operative device close under ultrasound-guidance.

The percutaneous occlusion has been considered the first choice for patients with secundum ASD, and it has been regarded as generally effective and safe [13, 14]. Good results are achievable not just for patient with smaller ASDs, but also for those with short aortic valve rim. However, the traditional intervention must be performed under fluoroscopic and guidance and has its limitations [15, 16]. They mainly include: (1) The radiation exposure can directly damage the body’s biological macromolecules, damage hematopoietic, endocrine, reproductive, and other systems, and may can induce malignant tumors; (2) X irradiation can also reduce the ability of the body to scavenge oxygen free radicals, and the body’s antioxidant function is in a state of inhibition, causing indirect damage. This is true especially for children under 10 years of age, where the sensitivity is significantly higher than that of adults, and the risk of radiation damage is significantly increased. (3) The operation time was prolonged, and the X-ray damage is serious when the size of ASD is big and the rim is small, so it is necessary to repeat the release procedure in a severe case. (4) The fluoroscopic imaging equipment is not accurate enough for the display of cardiac anatomy, especially in evaluate shunting. (5) Because younger children have smaller blood vessel diameters and cannot accommodate the delivery sheath, interventional closure is limited by age, especially those with large defects. It’s generally suitable for patients over the age of 3, and have the possibility of pericardial tamponade, iatrogenic pseudoaneurysm, and arteriovenous fistula: (6) Contrast agents may be used during surgery, which may impair kidney function.

Intra-operative device closure ASD is a new method of closure in recent years [5]. The right chest to right atrial route to closure ASD has the following advantages. First, under the guidance of TEE, medical workers and patients are protected from fluoroscopy damage. Second, real-time echocardiography monitor, which is clear for anatomical structure, can better observe and guide the occlusion device release process. Third, no age or weight limitation since the procedure is performed through a small right thoracotomy incision to access right atrium. Fourth, the delivery system is short and the technique is flexible. The delivery sheath tube is perpendicular to the atrial septal, it is easy to adjust the angle of the occluder release position, and the release is easier and more accurate. The procedure can be performed even if the size of ASD is large and the rim near the aortic valve or inferior vena cava is short or absent. Fifth, IODC was performed in the operating room. Once the device fails or procedure unsuccessful, it can be converted to conventional surgery with extracorporeal circulation. Under the IODC procedure, the danger to the patient during the delivery process is avoided. The disadvantage is that it must be performed under general anesthesia with tracheal intubation. It requires an incision of 2–3 cm in front of the right thoracic wall and has the possibility of damaging the internal mammary artery and affecting breast development [17].

Having a rim over 5 mm guarantees successful transthoracic occlusion. The rim’s shortest side is especially important for the stability of the occluder device, since the left side of the device slip into the right atrium often occurs at the shortest rim during release procedure [3]. At present, it is believed that the rim length of the ASD does not affect the success rate of occlusion. IODC procedure requires a larger rim on the superior vena cava and the inferior vena cava, while the rim of the aortic valve has exact demand. For ASDs with a short rim, a larger occluder was required [11].

Percutaneous ASD closure under TEE-guidance was performed by the puncture of a femoral vein without a significant surgical incision [18]. The whole process was completed under the guidance of TEE to avoid X-ray damage and further reduce the trauma. This study found that the size of the ASD, the rim of the inferior vena cava, and the criteria for selecting the occluder were important factors in the procedure success.

First of all, the size of the ASD is an important factor for the procedure’s success. In this study, the diameter of ASD was less than 25 mm. The percutaneous TEE-guided closure has a 100% success rate, but when the diameter of ASD more than 25 mm, the success rate is lower than that of IODC. Because during percutaneous closure, it is difficult to adjust the delivery sheath tip perpendicular to the atrial septum plane. The delivery sheath is kept an angle to the atrial septum, the occluder left disc cannot be parallel to the atrial septum, and the occluder will be slipping into left atrium during release the right side of device.

Secondly, the rim of the ASD is also very important for simple TEE-guided percutaneous closure, especially one near the vena cava. The inferior vena cava is required to be ≥5 mm. For the rim of the aortic valve, percutaneous occlusion is more demanding than IODC occlusion. Because the rim of the aortic valve serves as the fulcrum of the occluder’s upper edge, the rim of the aortic valve has a greater impact on percutaneous occlusion. Aortic valve rims of 3 mm or thicker increases the possibility of success.

Third, the size of the occluder is another key factor in successfully closeing the ASD. For a normal TEE-guided percutaneous ASD closure, the occluder size should be the maximum diameter of the defect plus 6 mm for the highest probability of success. Since the occluder is parallel to the interatrial septum during IODC, release is easy. When the ASD is less than 25 mm, the size of the occluder should be the longest diameter of defect plus 4 mm, or 4–6 mm if the longest diameter of the ASD is more than 25 mm.

Compared with IODC, TEE-guided percutaneous ASD closure is performed only under TEE guidance, avoiding x-ray irradiation [16, 19]. The difference is that it does not require a chest incision, less trauma, less scarring, and less time for surgery and hospital stay [20]. If operating under basic anesthesia or lidocaine without the need for endotracheal intubation, the patient also recovers faster [21]. However, the delivery sheath is long, and manipulation is difficult. Transthoracic closure is simpler because it is easier to adjust the delivery sheath to a position perpendicular to the interatrial septum, such that even if the rim of the aortic valve is close to zero the operation could be successful. For large diameter ASD defects, transcutaneous closure may be successful when transcutaneous closure fails [22]. Four patients in the PASD groups were transferred into IODC when device deployment failed. The rim of inferior vena cava not exceeding 3 mm was the critical factor, and all of them have successful results after change the surgical method.

## Limitation

In this study, the classic transcatheter closure ASD under DSA was not included in the control group, and the advantages of percutaneous closure of ASD under simple ultrasound guidance were only theoretically and empirically described. Moreover, a comparison with the transthoracic group was also not a randomized and controlled study. The number of cases enrolled in this study was still small, and the follow-up time was still short. In particular, the influence of the occluder on the aortic valve and the tricuspid valve after the procedure requires more cases and long-term follow-ups and observations.

## Conclusion

Atrial septal defect is the most common congenital heart disease in the clinic, and minimally invasive treatment is a great challenge for surgeons. Simple and minimally invasive ultrasound-guided, transthoracic occlusion is a surgical procedure that avoids both extracorporeal circulation and x-ray injury. Percutaneous ultrasound guided percutaneous ASD closure is safe and feasible for ASD where the maximum diameter is less than < 30 mm, resulting in less trauma, more visually pleasing with less scarring, and shorter hospital stays. For ASD with maximum diameters of more than 25 mm, having inferior vena cava rims of less than 3 mm is risky, and the IODC may be a better choice.

## Data Availability

The datasets used or analyzed during the current study are available from the corresponding author on reasonable request.

## Abbreviations

ASD: Atrial septal defects
CHD: Congestive heart defects
CPB: Cardiopulmonary bypass
IODC: Intra-operative device closure
TEE: Transesophageal echocardiography
TTE: Transthoracic echocardiography
